# Polygenic Risk Associations with Clinical Characteristics and Recurrence of Dupuytren Disease

**DOI:** 10.1097/PRS.0000000000010775

**Published:** 2023-05-31

**Authors:** Sophie A. Riesmeijer, Ilja M. Nolte, Loes M. Olde Loohuis, Lianne M. Reus, Toni Boltz, Michael Ng, Dominic Furniss, Paul M. N. Werker, Roel A. Ophoff

**Affiliations:** Groningen, Amsterdam, and Rotterdam, the Netherlands; Los Angeles, CA; and Oxford, United Kingdom; From the Departments of 1 Plastic Surgery; 2 Epidemiology, University of Groningen, University Medical Center Groningen; 3 Center for Neurobehavioral Genetics, Semel Institute for Neuroscience and Human Behavior, University of California, Los Angeles; 4 Alzheimer Center Amsterdam, Department of Neurology, Amsterdam Neuroscience, Vrije Universiteit Amsterdam, Amsterdam University Medical Center; 5 Nuffield Department of Orthopaedics, Rheumatology and Musculoskeletal Sciences, Botnar Research Centre, University of Oxford; 6 Department of Psychiatry, Erasmus University Rotterdam, Erasmus Medical Center.

## Abstract

**Background:**

Dupuytren disease (DD) is a common complex trait, with varying severity and incompletely understood cause. Genome-wide association studies (GWAS) have identified risk loci. In this article, we examine whether genetic risk profiles of DD in patients are associated with clinical variation and disease severity and with patient genetic risk profiles of genetically correlated traits, including body mass index (BMI), triglycerides, high-density lipoproteins, type 2 diabetes mellitus, and endophenotypes fasting glucose and glycated hemoglobin.

**Methods:**

The authors used a well-characterized cohort of 1461 DD patients with available phenotypic and genetic data. Phenotype data include age at onset, recurrence, and family history of disease. Polygenic risk scores (PRSs) of DD, BMI, triglycerides, high-density lipoprotein, type 2 diabetes, fasting glucose, and hemoglobin A1c using various significance thresholds were calculated with PRSice using the most recent GWAS summary statistics. Control data from LifeLines were used to determine *P* value cutoffs for PRS generation explaining most variance.

**Results:**

The PRS for DD was significantly associated with a positive family history for DD, age at onset, disease onset before the age of 50, and recurrence. We also found a significant negative correlation between the PRSs for DD and BMI.

**Conclusions:**

Although GWAS studies of DD are designed to identify genetic risk factors distinguishing case/control status, we show that the genetic risk profile for DD also explains part of its clinical variation and disease severity. The PRS may therefore aid in accurate prognostication, choosing initial treatment and in personalized medicine in the future.

**CLINICAL QUESTION/LEVEL OF EVIDENCE:**

Risk, III.

Clinical features and severity of Dupuytren disease (DD) vary substantially among patients: some have a solitary nonprogressive nodule throughout their lifetime, and others present with severe, recurrent flexion deformities bilaterally, accompanied by ectopic lesions and associated fibromatoses.^1^ Although its cause is not yet well understood, DD is a complex and highly heritable trait, with an estimated proportion of phenotypic variance attributable to common genetic variants [eg, single-nucleotide polymorphism (SNP) heritability] of 67%.^2,3^ An earlier age at onset is associated with increased severity, and a positive family history is associated with lower age at first surgery.^4^ Phenotypic associations have been observed between DD and diabetes mellitus, epilepsy, and liver disease.^2,5^ Genetically, DD shows positive correlations with body mass index (BMI), type 2 diabetes mellitus (T2D), triglycerides (TG), and high-density-lipoprotein (HDL), suggesting a shared heritable component.^3^ A recent genomewide association study (GWAS) has identified 26 genetic loci associated with DD, highlighting WNT signaling, extracellular matrix modulation, and inflammation to be involved in the pathogenesis of fibrosis.^6,7^ Together, these 26 loci explain 11.3% of the variance of DD. However, genetic markers below the genomewide significance threshold may still explain a considerable additional proportion of phenotypic variation.^7,8^

In this study, we set out to investigate whether genetic risk for DD could also explain disease severity, recurrence, and occurrence of associated disorders. To do so, we construct polygenic risk scores (PRSs) using the most recent GWAS of DD.^9^ In addition, we studied the correlation between PRSs of patients for DD and PRSs for BMI, TG, HDL, T2D, and endophenotypes fasting glucose (FG) and glycated hemoglobin (HbA1c) at various thresholds of statistical significance. We studied this correlation specifically in DD cases, as significant genetic correlations between GWAS results of these traits have previously been established (BMI, −0.196; TG, −0.139; HDL, 0.133; and T2D, −0.182).^3^ Thus, our goal was to further understand the nature of these correlations at different levels of association within well-characterized patient cohorts. In case of significant and strong association of DD clinical characteristics with the PRS, genetic profiling can in the future support personalized treatment strategies: DD patients are presently counseled based only on clinical characteristics, often before the full extent of their DD phenotype has become apparent. Understanding the contribution of genetic variants to patient characteristics and DD recurrence is a necessary step toward accurate prognostication of this highly heritable disease.

## PATIENTS AND METHODS

### Cohort Description

For this study, our previously described cohort of DD patients was used.^10,11^ Ethical approval for this study was obtained from the medical ethics committee of the University Medical Center Groningen, the Netherlands (2007/067). Briefly, phenotype and genotype data of total of 1669 DD patients were collected between 2007 and 2016. Phenotype data included data on clinometrics of flexion contractures; characteristics such as age at onset; family history (defined anamnestically as having at least one affected family member in the first or second degree); bilateral disease; ectopic disease; observed recurrence (defined as recurrence of flexion contracture or new signs of DD tissue in previously cleared areas); surgical recurrence (defined as two or more surgical interventions in the same ray); and the associated disorders diabetes mellitus, epilepsy, and liver disease. LifeLines is a multidisciplinary, prospective, population-based cohort study examining, in a unique three-generation design, the health and health-related behaviors of 167,729 persons living in the northern Netherlands.^12^ A detailed description of the LifeLines cohort genotype calling and quality control (QC) pipeline can be found on Github (https://github.com/molgenis/GAP).

### Genotyping and QC Procedures

Samples were genotyped in two batches using two different arrays, because of the longitudinal nature of our data collection. The first set of 960 DD cases and 15,638 LifeLines controls was genotyped using the Illumina HumanCytoSNP-12 array (CytoSNP) and called with Illumina’s Genome Studio. The second set included 709 DD cases and 36,339 LifeLines controls genotyped with the Illumina Global Screening Array (GSA) and called using Opticall.^13^ Cases and controls were genotyped separately.

QC was performed for CytoSNP and GSA releases separately using PLINK (version 1.9) and R (version 3.6.1).^14,15^ All markers were aligned to the positive strand and mapped to build GRCh37. Multiple variants were harmonized and removed in case of low concordance. SNPs from chromosomes X and Y and mitochondrial SNPs were removed.

Dupuytren cases and the LifeLines controls were genotyped separately, as they originated from separate studies. To reduce batch effects, for the GSA genotyping data we combined the raw data of probe intensities of all cases and 1200 random controls and called genotypes together using optiCall. The cases were next extracted from the calling data set.^13^ For the CytoSNP data, calling was done separately, because the raw data were not available anymore. We applied the QC pipelines of the respective control cohorts to QC of our case cohorts, adapting them for cases where necessary: first, we extracted only variants that survived QC from the control cohort from the respective genotyping platform. Second, the Hardy-Weinberg equilibrium *P* value threshold was released to 1 × 10^−10^. Lastly, allele frequencies of cases were compared with those of controls, and SNPs with an allele frequency that deviated (*P* < 1 × 10^−6^) were removed from the case data set. We hypothesized that this significance was more likely attributable to genotyping assay failures than true causality and expected true causal hits to be retrieved during imputation.

### Removal of Related Individuals

We used genomewide complex trait analysis to determine a set of unrelated LifeLines controls for both the CytoSNP and GSA array separately (pi-hat < 0.15).^14,16^ PLINK was next used to determine the relatedness between the LifeLines cohorts and duplicates or first- and second-degree relatives were removed from the CytoSNP data, because the GSA chip contains more markers (approximately 650,000 versus approximately 300,000).^14^ In addition, Dupuytren patients or their first- and second-degree relatives who also participated in the control cohort were removed from the latter.^14^

### Genotype Imputation

Imputation was next done using the 1000 genomes phase 1 for the CytoSNP cohorts and the Haplotype Reference Consortium as reference panel for the GSA cohorts for cases and controls separately using Sanger Imputation Server.^17–19^

### Merging of Case and Control Data Sets

As the mean age of DD cases was higher than that of controls, only controls with an age range similar to DD (mean, 62 years; interquartile range, 56 to 70 years) were selected. Imputed genotype data of cases and selected controls were merged for each genotyping release using BCFtools.^20^

### GWAS Summary Statistics

For all traits, summary statistics from the most recent GWASs in European ancestry individuals were used for PRS calculations.^3,7,19,21–24^ Summary statistics were either publicly available from the GWAS catalog or LD hub, or acquired through personal correspondence.^7,25,26^

### Polygenic Risk Scores

PRSs were constructed with the PRSice algorithm using default settings.^27^ These individual-level scores are calculated by summing the number of risk variants that a person carries weighted by effect sizes that are derived from an independent discovery GWAS, and usually include nongenomewide significant markers.^9^ One way to create PRSs is by clumping and thresholding: where independent SNPs are selected based on a *P* value threshold and several thresholds are tested to maximize predictive ability of the derived polygenic scores.^8^ Here, the optimal PRS was determined among a set of PRSs using *P* value thresholds ranging from *P* = 5 × 10^−8^ to *P* = 0.5 as the PRS that explained the most disease variance. For quantitative traits, we used the regression coefficient betas, and for binary traits, the log odds ratios as weights for the PRSs calculations.

Optimal PRSs were calculated for our two genotyping data sets (one with CytoSNP array and the other with GSA array genotype data) separately. We wished to combine the data of our two cohorts for a joint analysis. However, as different SNP arrays vary in genomewide genotyping density, which may affect successful genotype imputation, we regressed the optimal PRSs of each genotype release against their first five genotyping release-specific principal components (correcting for population structure),^14^ and standardized the residuals within each genotyping release before combining them into a standardized residualized PRS (ie, the optimal standardized residualized PRS, hereafter referred to as the PRS).

### Statistical Analyses

Quantile-quantile plots were produced to check the normality of the PRS distributions. Two-tailed *t* tests and chi-square tests were performed to test for differences in age and sex between genotyping releases, and for differences in the mean PRS between cases and controls.

Unrelated controls were selected from the LifeLines cohorts using GCTA and^14,16^ and used in the DD PRS analyses. The DD PRS analyses were performed with case/control status using univariate logistic regression. For T2D, TG, HDL, BMI, FG, and HbA1c, the PRS analyses were performed in only the control cohort using logistic or linear regression analysis, depending on the trait, as information on these phenotypes was not available in our case cohort. The optimal PRS for subsequent analysis was defined as the one with the highest Nagelkerke pseudo *R*^2^, which is a measure of proportion of phenotypic variance explained (goodness-of-fit) when applying different *P* value thresholds of GWAS results. The Nagelkerke *R*^2^ was adjusted to the liability scale using a population prevalence of DD. We used a previously estimated prevalence of DD that has been corrected for the general Dutch population of all ages of 7.08%.^28,29^ (**See Table, Supplemental Digital Content 1**, which shows the calculation of age-adjusted prevalence of DD in the general population of the northern part of the Netherlands, http://links.lww.com/PRS/G499.) For T2D, TG, HDL, BMI, FG, and HbA1c, for which only the population-based controls were used,^12^ no liability adjustment is applied. After establishing the PRSs explaining most of the phenotypic variance in controls, the PRSs at these optimal *P* value thresholds are calculated in the DD cases.

Univariate logistic regression analyses were next performed to test for the association between clinical characteristics and the PRS thresholds in DD cases, while adjusting for genotyping batch. We also performed a multivariable logistic regression analysis with the recurrence as outcome and the PRS and family history as predictors, to study the added value of the PRS over family history in predicting disease severity.

Because a study on cardiovascular risk demonstrated the 1% of patients with the highest PRS had a lifetime risk equivalent to the risk faced by those carrying monogenic Mendelian mutations, we similarly compared patients with the top decile PRSs to patients with the bottom decile PRSs.^30^ We chose the deciles because the number of patients in the top 1% was too small in our data set (*n* = 15).

Last, we calculated Pearson correlations between the PRSs for BMI, TG, HDL, T2D, FG, and HbA1c and the DD PRS for the optimal PRS threshold and thresholds 0.05 and 0.5 to observe differences in correlations between oligogenic and PRSs. Statistical analyses were performed in R (version 3.6.1).^15^ A Bonferroni correction was used to account for multiple testing resulting in a significance level of 0.05/13 = 0.0038 for association testing with 13 clinical characteristics. The Bonferroni correction for multiple testing is a means of accounting for the larger likelihood of finding a false-positive association when multiple comparisons are made simultaneously. For the Pearson correlations between the DD PRS and six-trait PRSs, a correction for multiple testing of 0.05/6 = 0.008 was used.

## RESULTS

### Cohort Description

During genotype QC, 43,038 variants, 75 cases, and 8101 controls were removed from the CytoSNP data set, and 113,556 variants, 39 cases, and 16,395 controls were removed from the GSA data set. After imputation, 5,417,839 variants, 885 cases, and 7321 controls remained in the CytoSNP data set, and 4,658,587 variants, 670 cases and 19,944 controls remained in the GSA data set. Clinical data were available for 823 of the 885 CytoSNP DD cases and 638 of the 670 GSA DD cases. The CytoSNP and GSA cohorts were not significantly different in distribution of sex and age (*P* = 0.097 and *P* = 0.193, respectively). (**See Table, Supplemental Digital Content 2**, which shows the clinical characteristics for CytoSNP cases and GSA cases, http://links.lww.com/PRS/G500.) An overview of clinical characteristics of all DD cases (CytoSNP and GSA combined) and controls used for the PRS calculation is shown in Table 1.

**Table 1.** Clinical Characteristics for Cases and Controls

Characteristic	DD Cases (%)	Controls (%)
Male sex	1099 (75.2)	7224 (42.7)
Age, yr		
Mean	62	61
Range	20–89	25–100^a^
Bilateral disease	623 (61.6)	NA^b^
Positive family history	735 (66.6)	NA^b^
Age at onset, yr		
Mean	55	
Range	13–85	NA^b^
Disease onset before the age of 50	530 (37.0)	NA^b^
No. of affected rays		
Median	1	
Interquartile range	0–2	NA^b^
Observed recurrence	224 (25.9)	NA^b^
Surgical recurrence	217 (32.6)	NA^b^
Doctor-reported ectopic disease	311 (34.5)	NA^b^
Patient-reported ectopic disease	640 (49.4)	NA^b^
Diabetes mellitus^b^	157 (11.4)	NA^b^

NA, not applicable.

a Age was acquired from the LifeLines baseline assessment collected between 2007 and 2013.

b Clinical characteristics of LifeLines controls were not associated with the standardized residualized PRS.

### Optimal *P* Value Threshold for the PRS Generation

**Fig. 1.**
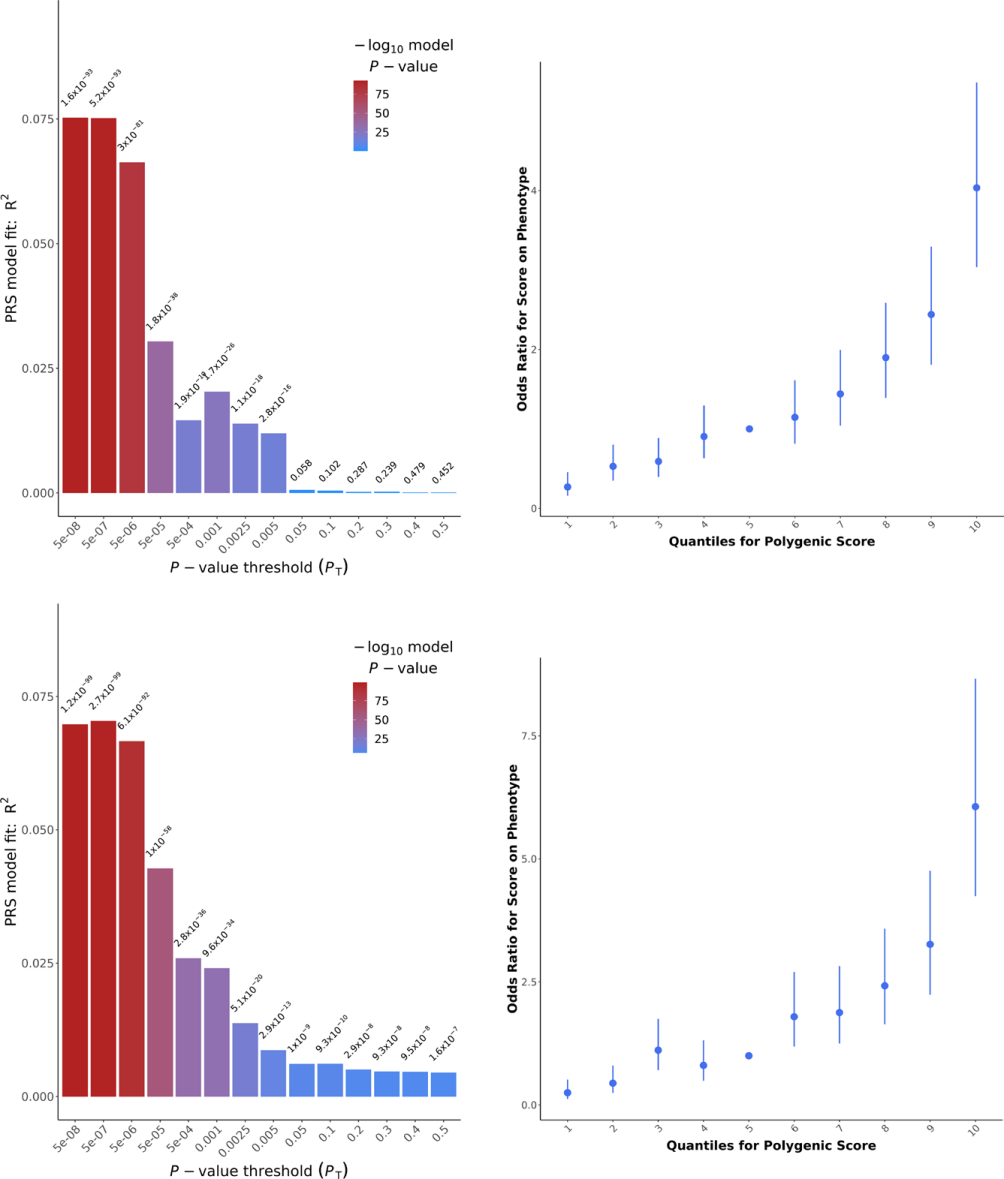
PRS analysis. (*Above*) Plots show the results for the CytoSNP data; (*below*) plots show the results for the GSA data. (*Left*) *Bar plots* with the explained variances for PRSs (*y* axis) composed of SNPs meeting different *P* value threshold (*x* axis). (*Right*) Odds ratios (including standard error bars) representing risk of DD for the optimal PRS (*P* value threshold of 5 × 10^−7^) per decile of the PRS.

The CytoSNP PRS analysis showed that the *P* value threshold 5 × 10^−8^ provided the PRS explaining most variance between cases and controls: 7.53% (Fig. 1). In the GSA, the *P* value threshold of 5 × 10^−7^ provides the optimal PRS with an explained variance of 7.04%. Because for the CytoSNP data, the variance explained at the 5 × 10^−7^ threshold was only 0.01% lower than at the 5 × 10^−8^, we chose to use the 5 × 10^−7^ threshold for both CytoSNP and GSA to homogenize the PRSs over both cohorts and to facilitate in combining them. The PRS at threshold 5 × 10^−7^ consisted of 81 independent variants available in both genotyping batches. [**See Figure, Supplemental Digital Content 3**, which shows (*first page*) distribution of optimal PRS per genotype release for DD cases; (*second page*) distribution of the standardized residualized optimal PRS for cases; (*third page*) distribution of optimal PRS per genotype release for controls; and (*fourth page*) distribution of the standardized residualized optimal PRS for controls, http://links.lww.com/PRS/G501.] We computed standardized residuals of the PRS as an independent variable and used genotyping platform as a covariate in regression analyses with clinical characteristics. As expected, the PRS differed significantly between cases and controls (*P* < 0.001). There were no significant differences in the residualized PRS between male and female subjects (*P* = 0.78), nor was there an association with age (*P* = 0.01, with α = 0.0038), when correcting for multiple testing. Liability-adjusted Nagelkerke pseudo *R*^2^ measures explained 4.5% and 16.9% of phenotypic variance in the CytoSNP and GSA cohorts, respectively.

### Association with Clinical Characteristics

**Fig. 2.**
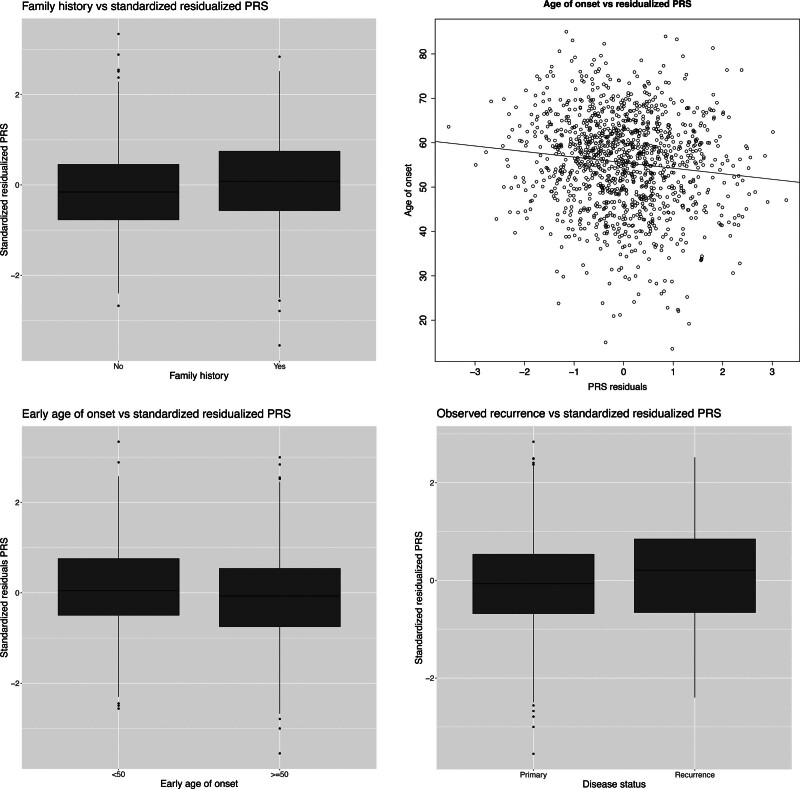
Association of the PRS for DD and (*above*, *left*) family history, (*above*, *right*) age at onset (continuous, including regression line), (*below*, *left*) disease onset before the age of 50 (below 50 years), and (*below*, *right*) observed recurrence.

Next, we examined the association between the PRS and clinical characteristics in the DD cases only (Table 2). Positive family history for DD, earlier age at onset, disease onset before the age of 50, and observed recurrence were significantly associated with a higher PRS, after correction for multiple testing (Fig. 2). Surgical recurrence and doctor- and patient-reported ectopic disease (Ledderhose disease, Peyronie disease, and knuckle pads), bilateral disease, number of affected rays, or diabetes mellitus were not significantly associated with the PRS after correction for multiple testing.

**Table 2.** Association of Clinical Characteristics with the Standardized Residualized PRS

Characteristic	No. of Cases in Analysis	Significance Level (*P*)	Effect Size^a^ (95% CI)	Liability Unadjusted Variance Explained (%)^b^
Bilateral disease	1012	0.87	0.99 (0.87–1.13)	—
Positive family history (yes/no)	1104	8.53 × 10^−4^^c^	1.24 (1.09–1.41)	27.0
Age at onset (continuous)	1213	4.31 × 10^−5^^c^	−1.25^d^ (−1.85 to −0.65)	—
Disease onset before the age of 50	1434	2.33 × 10^−4^^c^	1.23 (1.10–1.37)	28.2
No. of affected rays	1455	0.085	0.07^d^ (−0.01 to 0.16)	—
Observed recurrence	864	0.0035^c^	1.26 (1.08–1.47)	20.4
Surgical recurrence	667	0.022	1.22 (1.03–1.45)	—
Doctor reported ectopic disease	901	0.006	1.22 (1.06–1.40)	—
Patient reported ectopic disease	1295	0.021	1.14 (1.02–1.27)	—
Diabetes mellitus	1378	0.43	0.93 (0.79–1.10)	—

a Effect size is an odds ratio, unless otherwise indicated.

b Because the prevalence of the clinical characteristics in the general DD population is not well-documented in the literature, we did not perform liability adjustment of the variance explained.

c Statistically significant after Bonferroni correction (α = 0.05/13 = 0.0038).

d Regression coefficient beta (95% CI) instead of OR (95% CI) for linear regression.

In the model predicting observed recurrence with both the PRS and family history, the PRS (*P* = 0.01) showed a stronger association to observed recurrence than family history (*P* = 0.07), implying that the PRS seems to be a more powerful predictor of recurrence than family history.

### Extreme PRS and Clinical Characteristics

We compared clinical characteristics of individuals belonging to the highest decile of the PRS to those of individuals within the lowest decile of the PRS (*n* = 146 in each group). [**See Figure, Supplemental Digital Content 4**, which shows the proportion of clinical characteristics in the bottom and top deciles of the standardized residuals PRS. (*Above, left*) Family history. (*Above, right*) Age at onset. (*Second from top, left*) Early age at onset. (*Second from top, right*) Disease status. (*Center, left*) Bilateral disease. (*Center, right*) Number of affected rays. (*Second from bottom, left*) Surgical recurrence. (*Second from bottom, right*) Doctor-reported ectopic disease. (*Below, left*) Patient-reported ectopic disease. (*Below, right*) Diabetes mellitus. Occurrence of early age at onset (*P* = 0.001) and positive family history (*P* = 0.003) were significantly higher within cases belonging to the top 10% PRSs compared with those from the bottom 10% PRSs. PRS extremes were not associated with age at onset (*P* = 0.008), disease status (*P* = 0.021), occurrence of bilateral disease (*P* = 0.397), number of affected rays (*P* = 0.548), surgical recurrence (*P* = 0.245), ectopic disease (doctor- and patient-reported, *P* = 0.043 and *P* = 0.091 respectively), and diabetes mellitus (*P* = 0.812), after Bonferroni correction for multiple testing (α = 0.05/10 = 0.005), http://links.lww.com/PRS/G502.] Disease onset before the age of 50 and positive family history for DD were significantly different between the highest and lowest PRS deciles, after correction for multiple testing (α = 0.05/10 = 0.005) (*P* = 0.001 and *P* = 0.003, respectively). Cases within the highest PRS decile were at a 2.21 (95% CI, 1.36 to 3.62) and 2.47 (95% CI, 1.36 to 4.56) increased risk for a disease onset before the age of 50 and positive family history, respectively, compared with cases in the lowest decile. The PRS extremes were not significantly associated after correction for multiple testing with continuous age at onset (*P* = 0.008), observed recurrence (*P* = 0.021), doctor-reported ectopic disease (*P* = 0.043), occurrence of bilateral disease (*P* = 0.397), number of affected rays (*P* = 0.548), surgical recurrence (*P* = 0.245), patient-reported ectopic disease (*P* = 0.091), and diabetes mellitus (*P* = 0.812).

### Correlation the DD PRS and Other Traits’ PRSs

We found significant correlations between PRSs for DD and BMI at both *P* value threshold (0.05 and 0.5) (Table 3). The correlations of PRSs at 0.05 and 0.5 between DD and other traits were not statistically significant. Moreover, we found no significant correlations between the PRS for DD and other traits (Tables 4 and 5).

**Table 3.** Correlation of Standardized Residualized PRS of DD with PRS of BMI, TG, HDL, T2D, FG, and HbA1c at *P* Value Thresholds 0.05 and 0.5

Trait	PRS at 0.05		PRS at 0.5	
	Pearson Correlation Coefficient (95% CI)	Correlation *P*	Pearson Correlation Coefficient (95% CI)	Correlation *P*
BMI	−0.08 (−0.12 to −0.03)	0.003^a^	−0.10 (−0.14 to −0.05)	<0.001^a^
HDL	0.02 (−0.03 to 0.07)	0.42	0.02 (−0.03 to 0.07)	0.05
TG	−0.03 (−0.08 to 0.02)	0.32	−0.03 (−0.08 to 0.02)	0.29
T2D	−0.02 (−0.07 to 0.03)	0.42	−0.04 (−0.09 to 0.01)	0.09
FG	−0.06 (−0.011 to −0.01)	0.02	−0.03 (−0.08 to 0.02)	0.03
HbA1c	−0.01 (−0.06 to 0.04)	0.07	0.01 (−0.04 to 0.06)	0.59

a Statistically significant after Bonferroni correction (α = 0.05/6 = 0.008).

**Table 4.** *P* Value Thresholds of Optimal PRS for BMI, HDL, TG, T2D, FG, and HbA1c, Determined with LifeLines Controls

Disease	CytoSNP	GSA
BMI	0.05	0.1
HDL	5 × 10^−4^	2.5 × 10^−3^
TG	5 × 10^−4^	5 × 10^−6^
T2D	5 × 10^−3^	5 × 10^−5^
FG	5 × 10^−8^	5 × 10^−8^
HbA1c	5 × 10^−6^	5 × 10^−8^

**Table 5.** Correlation of Optimal Standardized Residualized PRS of DD with PRS of BMI, HDL, TG, T2D, FG, and HbA1c in DD Cases

Disease	Pearson Correlation Coefficient (95% CI)	*P*
BMI	−0.003 (−0.054 to 0.049)	0.918
HDL	0.034 (−0.017 to 0.085)	0.193
TG	0.028 (−0.024 to 0.079)	0.288
T2D	−0.004 (−0.056 to 0.047)	0.869
FG	0.014 (−0.07 to 0.066)	0.584

## DISCUSSION

Leveraging two large DD samples, we set out to test whether genetic risk for DD was also associated with clinical variation and disease severity. We found that multiple testing corrected significant association of the PRS with positive family history for DD, age at onset, early age at onset (before the age of 50), and observed recurrence. These results are in line with previous studies which significantly associated disease onset before the age of 50 and recurrence with family history or weighted genetic risk scores constructed with genomewide significant SNPs.^4,11^ Harmonious with these previous studies, we also found nominally significant associations (*P* < 0.05) of the PRS with surgical recurrence and doctor- and patient-reported ectopic disease. Although these characteristics were not significantly associated with PRS after stringent correction for multiple testing, these findings could indicate a potential effect of the PRS on these clinical characteristics. Bonferroni correction is a slightly conservative method that increases the chances of falsely rejecting truly significant associations, as it assumes that the different tests performed are independent.^31^ However, larger sample sizes are needed to confirm these associations. In the present study, we did not find an association of the PRS with bilateral disease, contrary to previous studies. This difference in results could be explained by the larger power of the present study through (1) using a continuous PRS for association testing, instead of categorical one or dichotomous family history; (2) using a larger study cohort; and (3) using a PRS that included over three times more variants, explaining more genetic variance than a weighted genetic risk score that includes only genomewide significant SNPs.

PRSs are a function of the GWAS sample size from which the weights are estimated.^8,32^ Performing a meta-analysis of all DD GWAS studies available to date could increase the accuracy of gene effects and thus add to the predictive accuracy of future PRSs. However, the facts that the heritability of DD is large (approximately 80%), the amount of trait variance explained by genomewide significant SNPs (11.3%) is moderate, and the optimal PRS (explaining most phenotypic variance) for GWAS results had a cutoff of *P* = 1 × 10^−7^, show that known genetic risk loci for DD explain quite a lot of the disease variance of DD already. In fact, the PRS outperformed family history in predicting observed recurrence in our multiple logistic regression including both the PRS and family history. Although family history is able to also capture shared nongenetic factors, a PRS can capture (naturally occurring) individual genetic variation within families that shared family history cannot.^32^ Thus, the PRS can be viewed as a more objective predictor of genetic predisposition than family history.^32^ Furthermore, PRSs can also be used for cross-trait prediction.^9,32^

The PRS for DD showed a strong goodness-of-fit for DD case-control status variance at lower *P* value thresholds (Fig. 1). The optimal PRSs were constructed from only 81 independent genetic variants reaching a *P* value threshold of 5 × 10^−7^, and the PRSs performed poorly at higher *P* value thresholds. One explanation for this observation is that DD has an oligogenic rather than a polygenic nature. This could make functional studies particularly fruitful.^3^ When correlating the PRSs for DD to those for BMI, TG, HDL, T2D, FG, and HbA1c at *P* value thresholds 0.05 and 0.5, BMI did in fact show a significant correlation to DD, meaning that the shared genetic cause of these traits is not driven by the genetic risk variants included in the oligogenic risk range for DD but by a much broader bandwidth of genetic variants.

The variance of DD occurrence explained by the PRS (liability-adjusted Nagelkerke pseudo *R*^2^), in the CytoSNP cohort was relatively low (4.5%) but in the GSA cohort, quite large (16.9%). We cannot fully explain this difference in variance explained; however, it may be attributable to the denser SNP coverage of the GSA genotyping array and thus higher imputation quality in the GSA cohort. Another explanation is that the patient populations differed more than expected.

The strengths of this study include a large study population with extensive phenotype data and the use of PRSs that explain more variance than previously used weighted genetic risk scores using only genomewide loci, increasing their accuracy. A limitation of our study was that, for determining the optimal PRS, we used controls that were not genotyped in the same batch as the cases. To reduce batch effects and affirm valid study results, we pursued a more stringent QC than traditionally done for a GWAS. Another limitation was the use of retrospective data. A prospective research design can increase quality of clinical characterization and classification of recurrence (eg, recurrence based on return of extension deficit, recurrence of DD tissue, or reoperation). This would also enable prediction modeling studies, in particular focusing on the identification of subgroups of DD patients (eg, to evaluate the association between genetic risk scores and response to treatment or time to recurrence after treatment). Genetic investigations into risk of recurrence and time to recurrence are a next step forward in the future of possibly implementing genetic risk prediction in personalized medicine for DD. If the PRS is a significant and strong predictor of recurrence and time to recurrence, assessment of patients’ PRSs could—on top of demographic and clinical factors—aid in clinical decision-making for the hospital population of DD patients.^33^ Estimation of recurrence risk and subsequent patient management are currently based on diathesis characteristics. However, some diathesis characteristics (eg, bilateral disease and ectopic disease) might not yet have become apparent at presentation, as opposed to a PRS, which can be determined at patient presentation. PRSs might contribute to a more timely and more accurate choice of timing and extent of treatment, choosing a more invasive approach for patients with a high risk for recurrence and a more hesitant (wait-and-see) approach in patients at a low risk for recurrence.

## DISCLOSURE

Dr. Werker was a member of a safety and efficacy review board for Fidia Ltd, Milan, in the development of new collagenase. None of the authors has a financial interest in the work presented in this article.

## DISCLAIMER

The views expressed in this article are those of the authors and not necessarily those of the National Health Service, the National Institute for Health Research, or the Department of Health.

## ACKNOWLEDGMENTS

This research subject to this study was funded by the University Medical Center Groningen, the Netherlands. The LifeLines initiative has been made possible by subsidy from the Dutch Ministry of Health, Welfare, and Sport; the Dutch Ministry of Economic Affairs; the University Medical Center Groningen, Groningen University; and the provinces in the northern Netherlands (Drenthe, Friesland, and Groningen). Dr. Riesmeijer was supported by the De Cock Foundation, Groningen, the Netherlands, for travel and housing expenses during her stay in Los Angeles, California. Dr. Reus has been supported by a personal Alzheimer Nederland fellowship, called “Genetic and Functional Overlap between Behavioural Variant Frontotemporal Dementia and Psychiatric Disorders” (WE.15-2018-11). Research of the Alzheimer Center Amsterdam (to L.M.R.) is part of the neurodegeneration research program of Amsterdam Neuroscience. The Alzheimer Center Amsterdam is supported by the Stichting Alzheimer Nederland and Stichting VUmc funds. The research of the Botnar Research Centre (to D.F.) was supported by the National Institute for Health Research, Oxford Biomedical Research Centre.

## Supplementary Material
