# Supplementary material for: Polygenic Risk Associations with Clinical Characteristics and Recurrence of Dupuytren Disease
Source: Plast Reconstr Surg. 2023 May 31;153(3):573–83. doi: 10.1097/PRS.0000000000010775 (PMC10876167; doi:10.1097/PRS.0000000000010775)
Supplement: Supplementary file 1 [file prs-153-573e-s001.pdf]

Supplemental Table. Calculation of age-adjusted prevalence of DD in the general population of the northern part of the Netherlands.

Age categories (years)	Mean proportion of prevalence per age category*	Proportion of people per age category of the general population†	Mean proportion of prevalence weighted by age category
**0-10**	0.001	0.125	0.0001
**10-20**	0.001	0.119	0.0001
**20-30**	0.001	0.135	0.0001
**30-40**	0.010	0.166	0.0017
**40-50**	0.040	0.148	0.0059
**50-60**	0.120	0.126	0.0152
**60-70**	0.200	0.086	0.0172
**70-80**	0.290	0.064	0.0184
**80-90**	0.370	0.027	0.0101
**>90**	0.450	0.004	0.0019
**Total**	**-**	**1.00**	**0.0708**

* From Figure 2, Lanting et al.(10)

† From Statistics Netherlands.(11)
