# Supplementary material for: Polygenic Risk Associations with Clinical Characteristics and Recurrence of Dupuytren Disease
Source: Plast Reconstr Surg. 2023 May 31;153(3):573–83. doi: 10.1097/PRS.0000000000010775 (PMC10876167; doi:10.1097/PRS.0000000000010775)
Supplement: Supplementary file 2 [file prs-153-573e-s002.pdf]

Supplemental Table. Clinical characteristics for CytoSNP cases and GSA cases.

Clinical characteristic	CytoSNP	GSA
**Male sex (n (%))**	625 (76.9%)	357 (73.6%)
**Age (mean [range])**	63 [24-88]	63 [20-89]
**Bilateral disease (n (%))**	296 (59.0%)	330 (64.3%)
**Positive family history (n (%))**	404 (65.0%)	332 (68.5%)
**Age of onset (mean [range])**	55 [15-85]	55 [13-83]
**Disease onset before the age of 50 (n (%))**	293 (36.4%)	240 (37.9%)
**Number of affected rays (median[IQR])**	1 [0-1]	1 [0-2]
**Observed recurrence (n (%))**	104 (26.9%)	122 (25.4%)
**Surgical recurrence (n (%))**	165 (30.1%)	53 (44.5%)
**Doctor reported ectopic disease (n (%))**	143 (29.9%)	170 (40.0%)
**Patient reported ectopic disease (n (%))**	347 (45.3%)	297 (55.7%)
**Diabetes mellitus (n (%))†**	97 (12.7%)	60 (9.7%)

IQR, inter quartile range
