# Supplementary figures and images for: Polygenic Risk Associations with Clinical Characteristics and Recurrence of Dupuytren Disease
Source: Plast Reconstr Surg. 2023 May 31;153(3):573–83. doi: 10.1097/PRS.0000000000010775 (PMC10876167; doi:10.1097/PRS.0000000000010775)

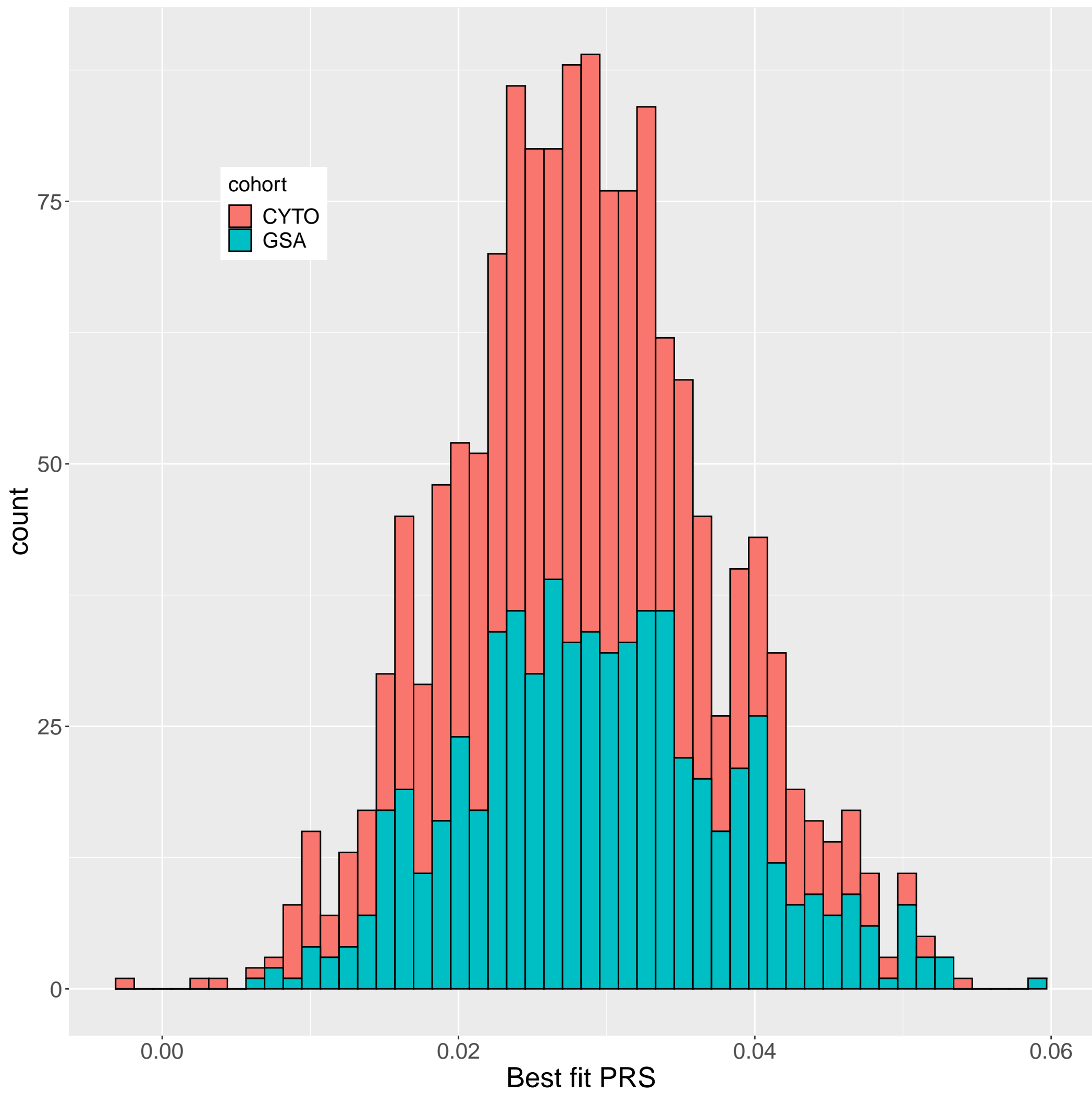

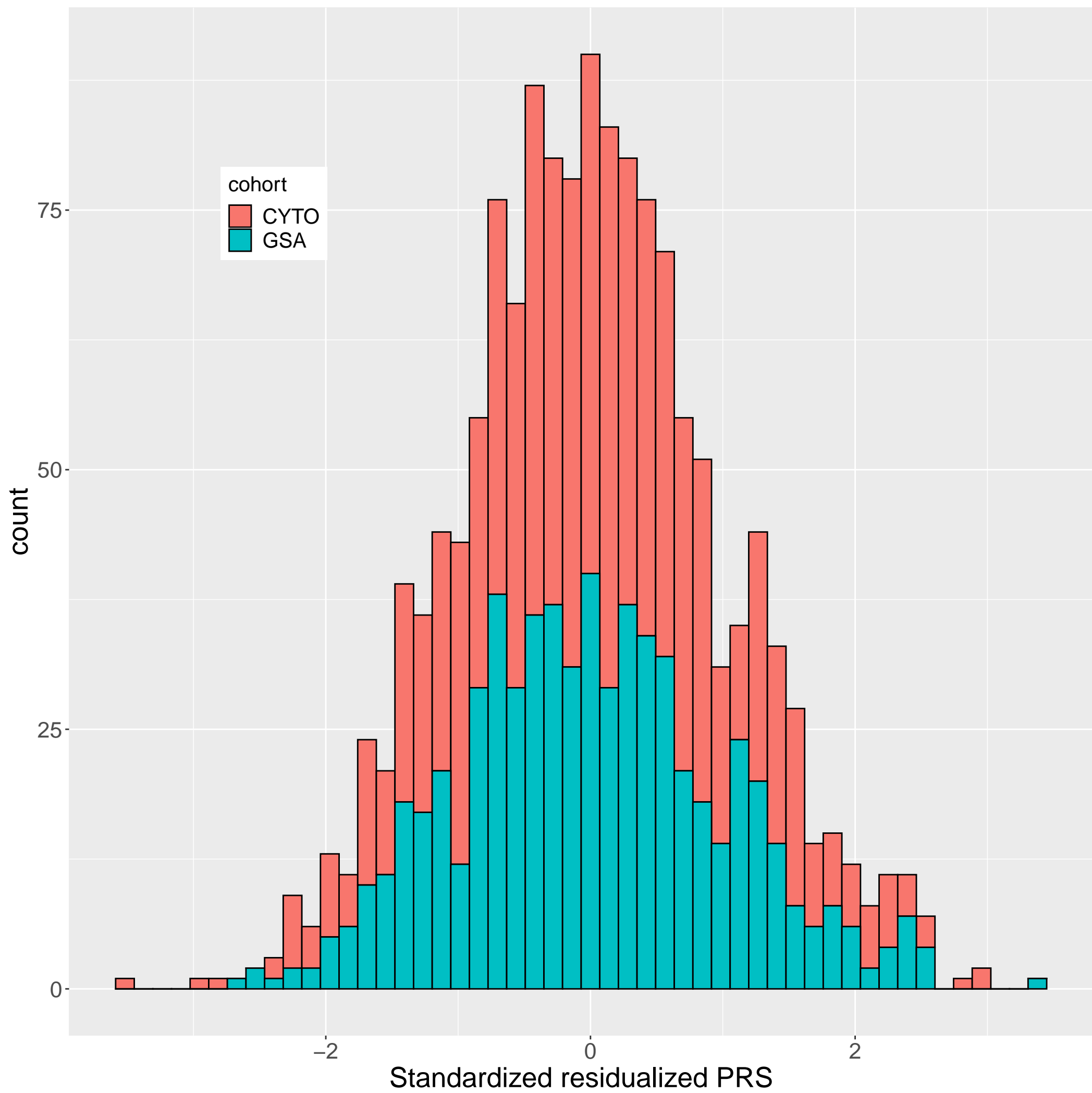

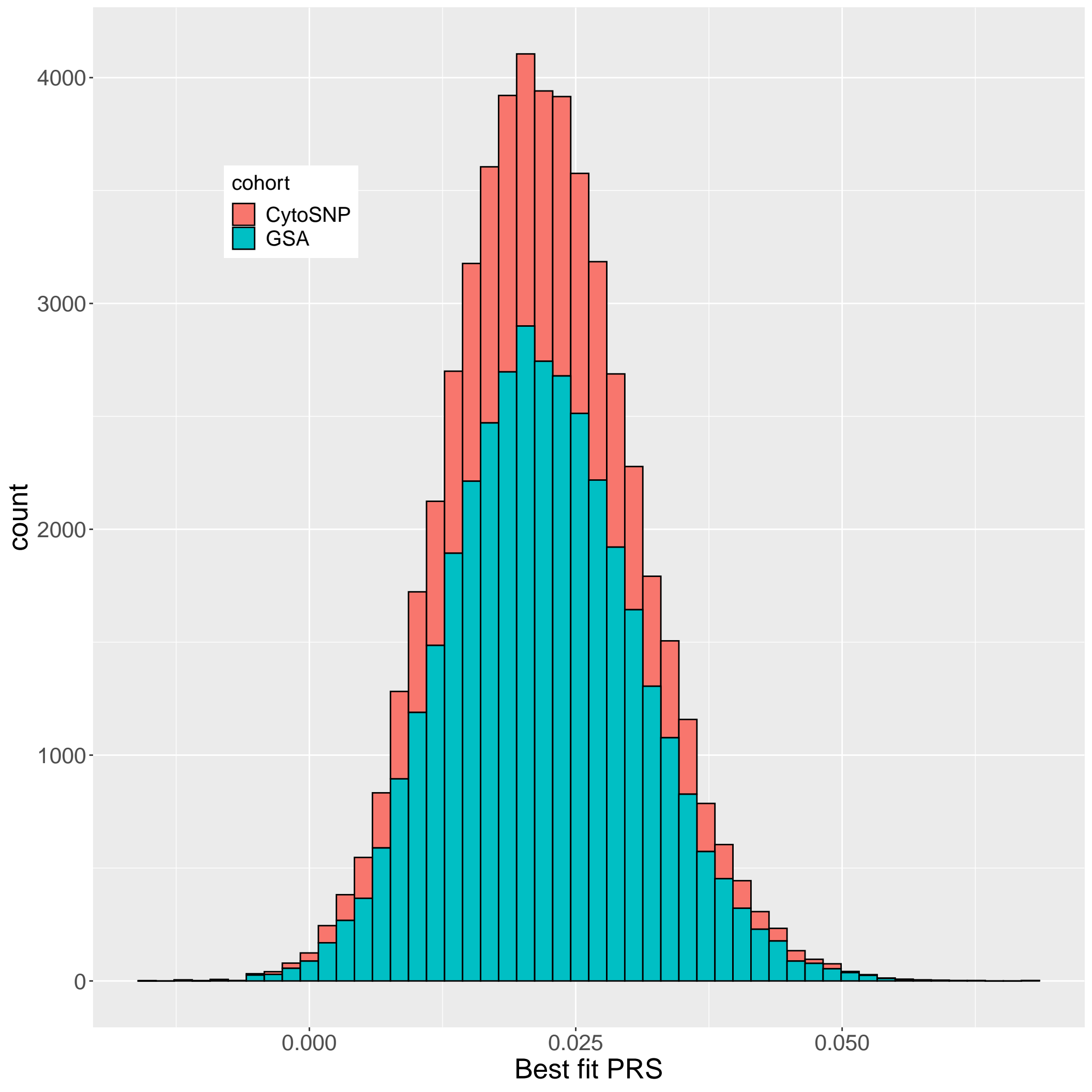

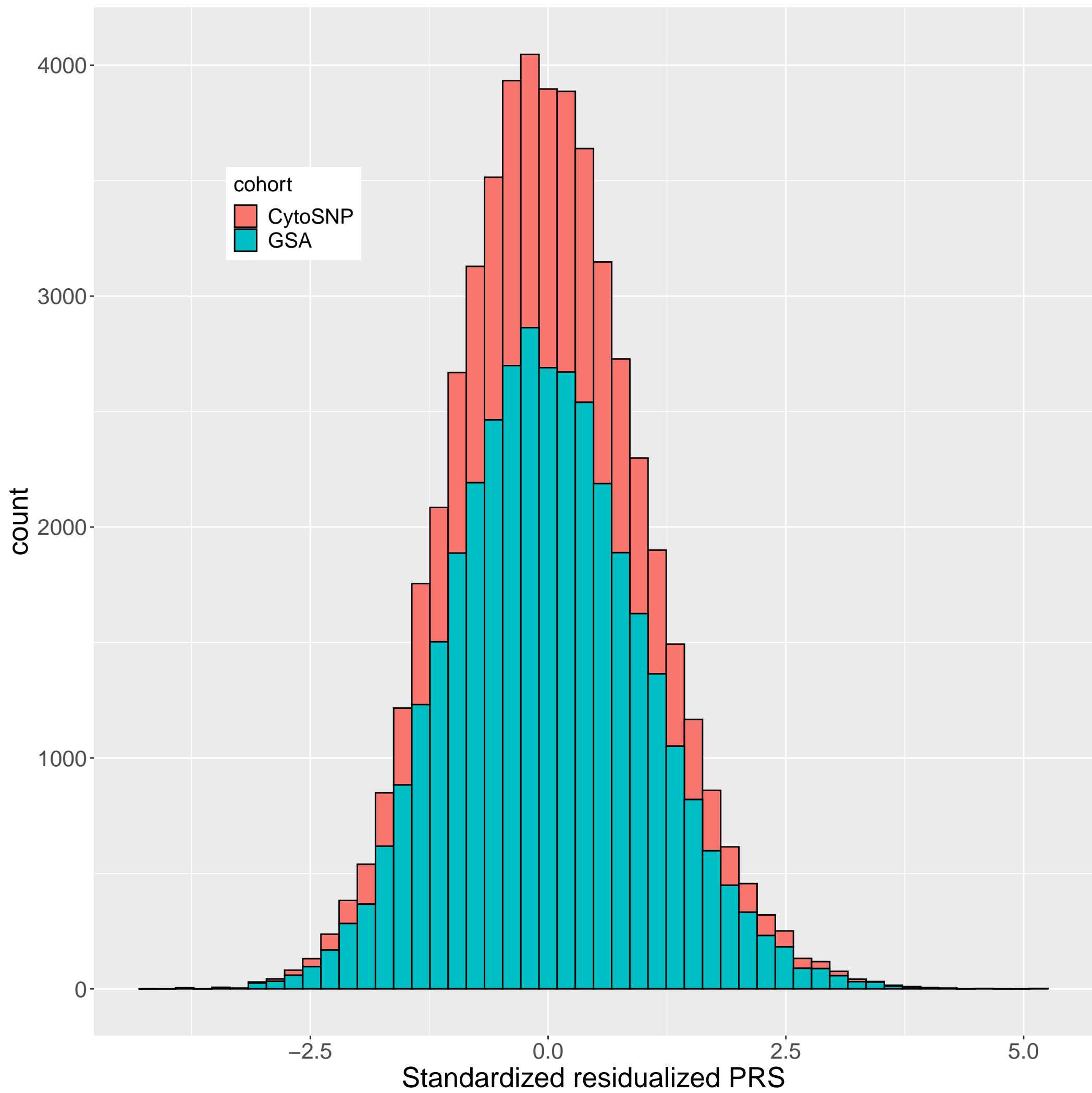

Supplement: Supplementary file 3 [file prs-153-573e-s003.pdf]

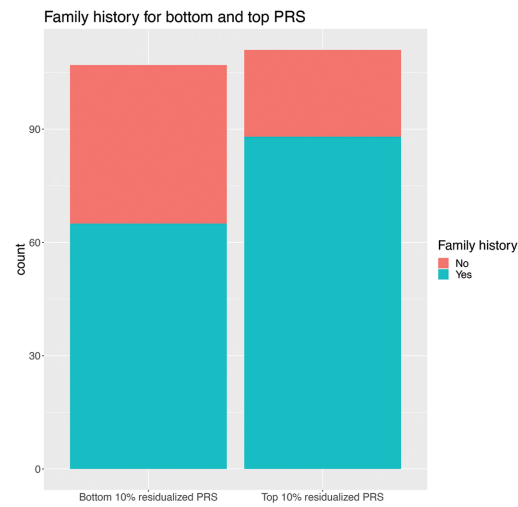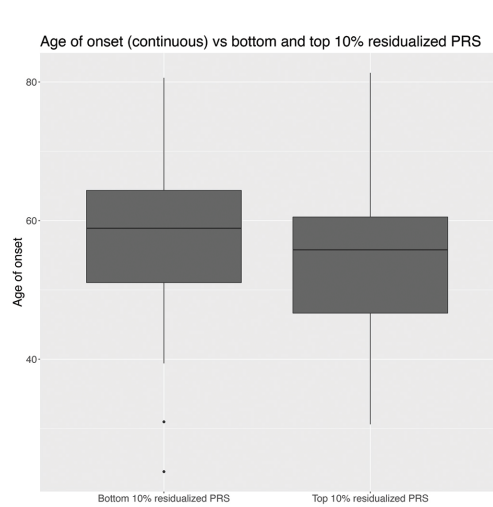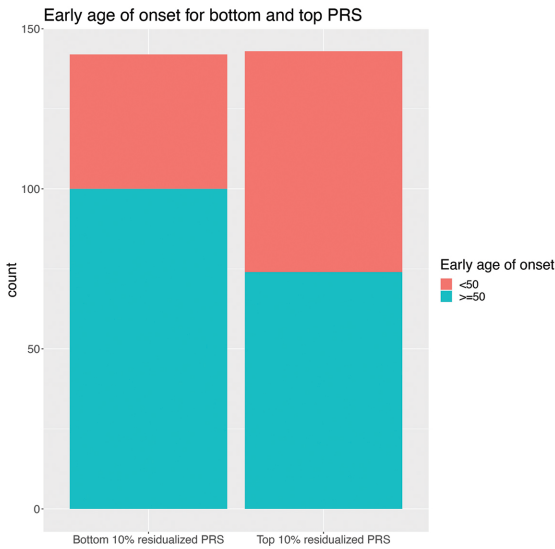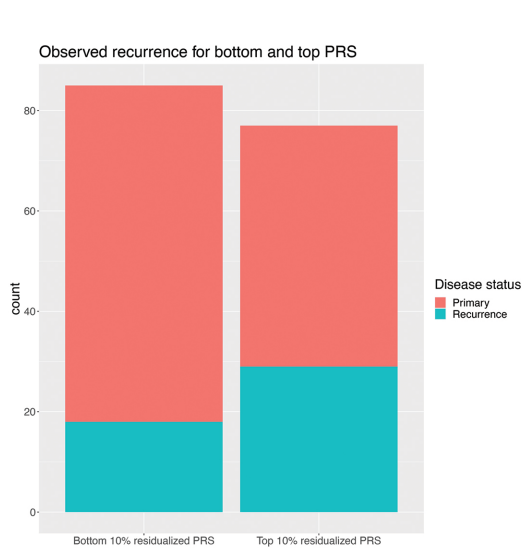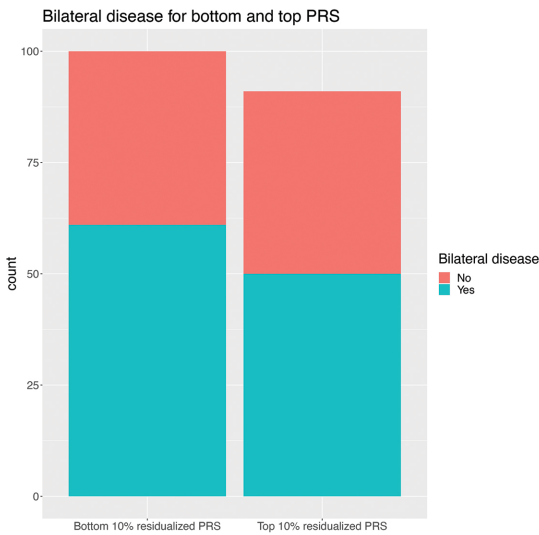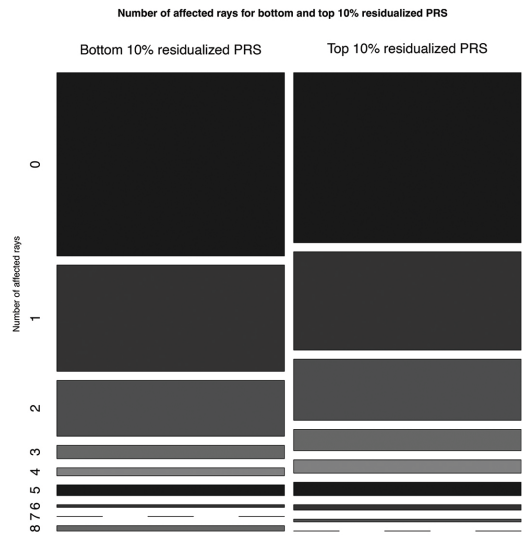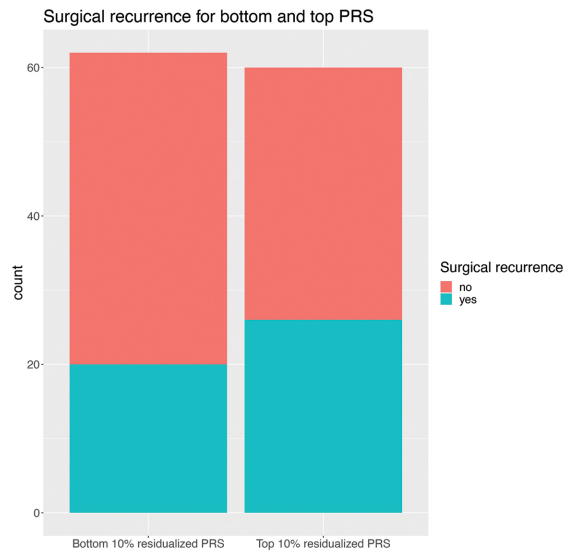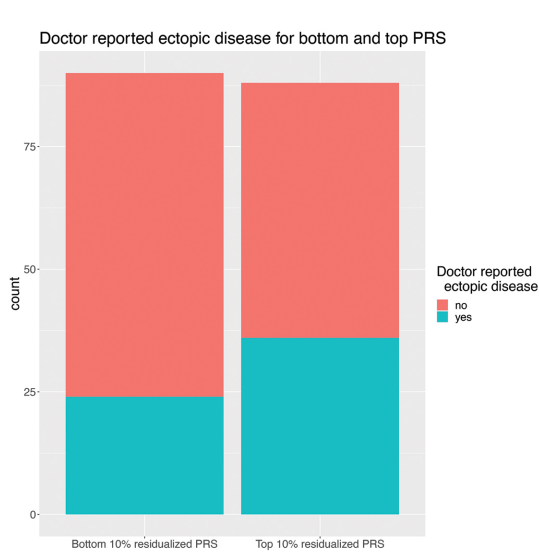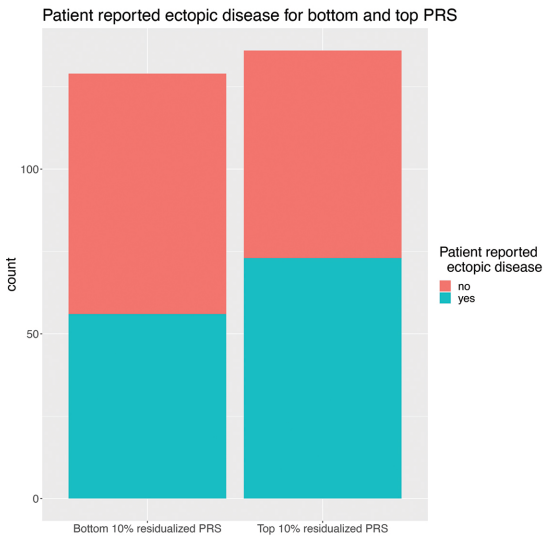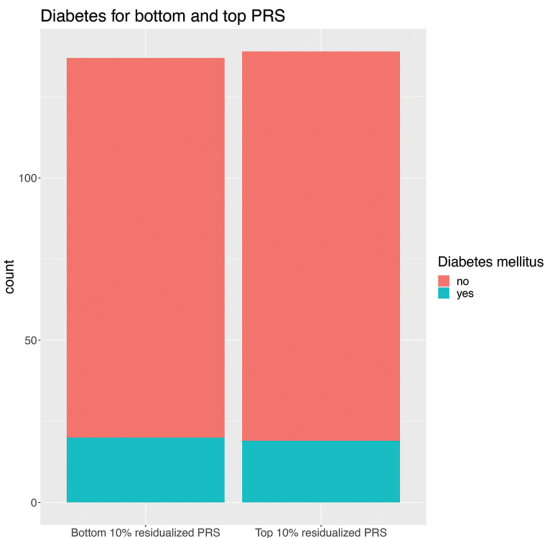

Supplement: Supplementary file 4 [file prs-153-573e-s004.pdf]
